# Non-invasive evaluation of muscle disease in the canine model of Duchenne muscular dystrophy by electrical impedance myography

**DOI:** 10.1371/journal.pone.0173557

**Published:** 2017-03-24

**Authors:** Chady H. Hakim, Alex Mijailovic, Thais B. Lessa, Joan R. Coates, Carmen Shin, Seward B. Rutkove, Dongsheng Duan

**Affiliations:** 1 Department of Molecular Microbiology and Immunology, School of Medicine, The University of Missouri, Columbia, MO, United States of America; 2 Department of Neurology, Beth Israel Deaconess Medical Center, Boston, MA, United States of America; 3 Department of Veterinary Medicine and Surgery, College of Veterinary Medicine, University of Missouri, Columbia, MO, United States of America; 4 Department of Biomedical Sciences, College of Veterinary Medicine, University of Missouri, Columbia, MO, United States of America; 5 Department of Neurology, School of Medicine, University of Missouri, Columbia, MO, United States of America; 6 Department of Bioengineering, University of Missouri, Columbia, MO, United States of America; University of Edinburgh, UNITED KINGDOM

## Abstract

Dystrophin-deficient dogs are by far the best available large animal models for Duchenne muscular dystrophy (DMD), the most common lethal childhood muscle degenerative disease. The use of the canine DMD model in basic disease mechanism research and translational studies will be greatly enhanced with the development of reliable outcome measures. Electrical impedance myography (EIM) is a non-invasive painless procedure that provides quantitative data relating to muscle composition and histology. EIM has been extensively used in neuromuscular disease research in both human patients and rodent models. Recent studies suggest that EIM may represent a highly reliable and convenient outcome measure in DMD patients and the mdx mouse model of DMD. To determine whether EIM can be used as a biomarker of disease severity in the canine model, we performed the assay in fourteen young (~6.6-m-old; 6 normal and 8 affected) and ten mature (~16.9-m-old; 4 normal and 6 affected) dogs of mixed background breeds. EIM was well tolerated with good inter-rater reliability. Affected dogs showed higher resistance, lower reactance and phase. The difference became more straightforward in mature dogs. Importantly, we observed a statistically significant correlation between the EIM data and muscle fibrosis. Our results suggest that EIM is a valuable objective measurement in the canine DMD model.

## Introduction

Duchenne muscular dystrophy (DMD) is a devastating degenerative muscle disease affecting 1 in every 5,000 male births [1–3]. DMD is caused by the loss of dystrophin, an essential muscle structure protein. Absence of dystrophin renders muscle susceptible to contraction-induced damage and eventually muscle death and fibrosis. Over the last three decades, tremendous information has been generated regarding disease mechanisms and experimental therapeutics using inbreed mouse models, in particular the mdx mouse [4]. Unfortunately, translation of the mdx data to human patients has been modest due to the limitation of the model [5–7]. Standard C57BL/10-background mdx mice do not develop clinical disease as seen in DMD patients [4]. Dystrophin-deficient dogs, on the other hand, show characteristic symptoms of muscular dystrophy and they also have a body size closer to that of humans [4]. Clearly, results obtained from dystrophic dogs will better inform the design of future clinical studies [8, 9].

Despite the general appreciation of the dog model, our understanding of dystrophic dogs remains limited [4, 10, 11]. A particular challenge is the lack of reliable, easy to use and non-invasive assays to monitor disease progression and response to therapy. Muscle biopsy, magnetic resonance imaging (MRI) and force measurement require putting affected dogs under general anesthesia which poses a significant risk of sudden cardiac death, malignant hyperthermia and rhabdomyolysis [12–16]. Gait analysis and activity monitoring are good non-invasive whole body assays, but they cannot provide disease status of individual muscle [17, 18].

Electrical impedance myography (EIM) is a painless, non-invasive, portable and easy to use technique to assess intrinsic muscle electric properties [19]. In EIM, a weak, high frequency electrical current is passed between two outer electrodes and the resulting voltages are measured from two inner electrodes. The electric impedance signals are determined by muscle composition, texture and architecture (such as myofiber size, edema, fatty infiltration and fibrosis). The EIM data allow investigators to quantitatively analyze muscle composition and structure. EIM has been extensively used as a painless and reliable outcome measurement to study various neuromuscular diseases including amyotrophic lateral sclerosis (ALS) [20–27], spinal muscular atrophy [28], facioscapulohumeral muscular dystrophy [29], congenital muscular dystrophy [30], inflammatory myopathy [31], inclusion-body myositis [19, 32], radiculopathy [33], and disuse atrophy [34]. Of relevance to our study, EIM has been successfully used in DMD patients and mdx mice [35–42]. These studies have revealed excellent reliability and validity of EIM as a powerful noninvasive biomarker for both pre-clinical and clinical studies.

Here we evaluated for the first time whether EIM can be used to distinguish muscle status in normal and affected dogs at ~ 6.6-m-old (young) and ~ 16.9-m-old (mature). We found EIM is a highly reliable and easy to use assay to study skeletal muscle in conscious dogs. Clear differences were detected between normal and affected dogs in multiple EIM parameters. Further, EIM changes correlated with the amount of fibrotic tissue in dog muscle.

## Materials and methods

### Animals

All animal experiments were approved by the Animal Care and Use Committee of the University of Missouri and were performed in accordance with NIH guidelines. A total of 24 dogs were used in the study including 10 normal male dogs and 14 affected dogs of both sexes (Table 1). Of the normal dogs, five were at the ages between 5.7 and 7-month-old (young normal dogs) and four were at the ages between 16.1 and 19.1-month-old (mature normal dogs). Of the affected dogs, eight were at the ages between 5.7 and 8.6-month-old (young affected dogs) and six were at the ages between 16.1 and 16.7-month-old (mature affected dogs). All experimental dogs were on a mixed genetic background and generated in house by artificial insemination. Affected dogs carry various mutations in the dystrophin gene that abort dystrophin expression. The genotype was determined by polymerase chain reaction according to published protocols [43–45]. The diagnosis was confirmed by the significantly elevated serum creatine kinase (CK) level in affected dogs. In a subset of dogs, diagnosis was also confirmed by muscle biopsy. All experimental dogs were housed in a specific-pathogen free animal care facility and kept under a 12-hour light/12-hour dark cycle. Affected dogs were housed in a raised platform kennel while normal dogs were housed in regular floor kennel. Depending on the age and size, two or more dogs are housed together to promote socialization. Normal dogs were fed dry Purina Lab Diet 5006 while affected dogs were fed wet Purina Proplan Puppy food. Dogs were given *ad libitum* access to clean drinking water. Toys were allowed in the kennel with dogs for enrichment. Dogs were monitored daily by the caregiver for overall health condition and activity. A full physical examination was performed by the veterinarian from the Office of Animal Research at the University of Missouri for any unusual changes (such as behavior, activity, food and water consumption, and clinical symptoms). The body weight of the dogs was measured every two weeks to monitor growth. Blood biochemistry was evaluated every 3 months in the first year and every six months thereafter. None of the experimental subjects were euthanized at the end of this study. However, a protocol was in place for euthanasia of animals at humane endpoints according to the 2013 AVMA Guidelines for the Euthanasia of Animals. Clinical signs for early euthanasia may include inability to obtain feed or water, pain unresponsive to analgesic therapy, paralysis of one or more extremities, and other signs of severe organ system dysfunction non-responsive to treatment or with a poor prognosis as determined by the veterinarian.

**Table 1:** Demographic information of experimental dogs.

Dog ID	Age (m)	Type*	Sex	BW (kg)	Biopsy
E06	5.7	N	M	19.2	No
E21	5.7	N	M	20.0	Yes
E09	5.7	N	M	15.9	Yes**
E15	5.7	N	M	19.2	No
E11	7.0	N	M	19.5	Yes
E14	7.0	N	M	20.5	Yes
E01	5.7	A	F	10.2	No
E02	5.7	A	F	8.2	No
E13	7.0	A	F	18.6	Yes
E18	7.0	A	F	9.8	No
E04	7.1	A	M	16.2	No
E07	7.4	A	M	16.6	Yes
E10	7.4	A	F	10.4	No
E08	8.6	A	F	13.1	Yes
E03	16.1	N	M	19.0	No
E12	16.1	N	M	21.2	Yes
E24	19.1	N	M	17.2	No
E23	19.1	N	M	18.1	Yes
E20	16.1	A	M	19.0	No
E22	16.1	A	M	21.7	No
E17	16.1	A	M	15.1	Yes
E16	16.6	A	F	20.2	No
E19	16.6	A	F	17.1	No
E05	16.7	A	M	20.4	Yes

*, Type refers to the genotype of the dog. N stands for a normal dog and A stands for an affected dog.

**, Hydroxyproline assay was not performed due to insufficient amount of tissue obtained from biopsy.

### EIM measurements

**Fig 1:**
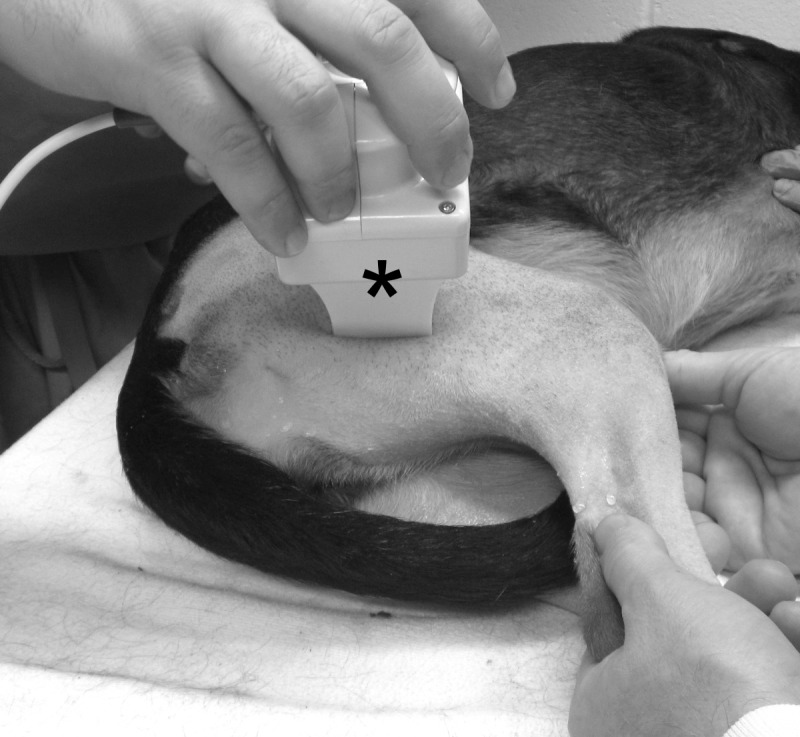
EIM assessment in dogs. The photomicrograph illustrates the placement of the EIM device on the biceps femoris muscle of an experimental subject. Asterisk, the EIM device used in the assay.

The handheld EIM 1103 device (Skulpt, Inc., San Francisco, CA) was used in the assay to collect multi-frequency impedance data (Fig 1). This device measures amplified signals directly using high-speed analog-to-digital converters. The EIM 1103 has a disposable multi-electrode array held in place via magnets. The electrode array used in this study was originally designed for use in young children and consists of three groups of electrodes, two nested sets for assessing current flow parallel to the major muscle fiber direction and one set for transverse measurement. The nested electrode design provides different depths of current penetration.

We chose to study the biceps femoris muscle because (1) it was sufficiently large to accommodate the EIM electrode array, which was developed for use in pediatric patients, (2) it was one of the most convenient muscles to perform the assay, and (3) it is the muscle most commonly used in biopsy.

The dog was awake throughout the assay and gently restrained in lateral recumbency (Fig 1). The muscle was identified according to anatomic markings. The fur on the skin was carefully removed with a fine shaving razor to ensure the skin was smooth for good contact with the electrode. The skin was cleaned and moistened with physiological saline. The electrode surface of the EIM device was positioned over the center of the bulk of the muscle on the skin as described in detail elsewhere [46]. Three consecutive multi-frequency impedance measurements were made on each muscle. Each measurement was performed at 40 discrete frequencies between 1 kHz to 1 MHz. The surface voltage patterns were recorded by the EIM device. The skin was remoistened with saline between each individual measurement. For each measurement, we collected data from three different electrode array configurations including the short longitudinal array, short transverse array and long longitudinal array. The real and imaginary components of the impedance (resistance and reactance) were calculated from the recorded voltages [19]. The phase was derived from the ratio of reactance and resistance. Specifically, phase (in radians) = arctan (reactance/resistance). The resulting value in radians is then converted to degrees by multiplying the conversion constant 57.296. The assay was performed on both the left and right side in each dog.

**Fig 2:**
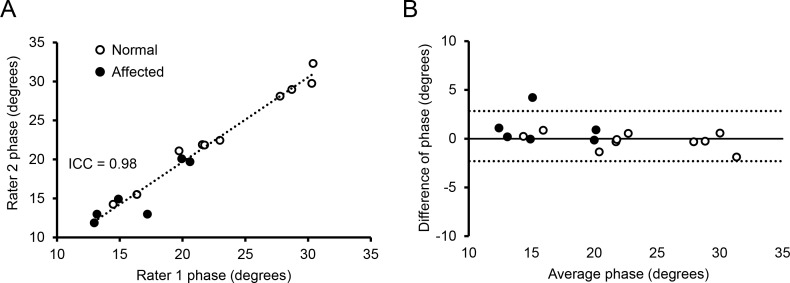
Inter-rater reliability of the EIM assay in normal and affected dogs. (A) The intra-class correlation plot of the two evaluators for phase at 150 kHz. (B) The Bland–Altman inter-rater plot. The solid line indicates the mean difference. Dashed lines mark standard deviations. Open circle, normal dogs; closed circle, affected dogs. Each circle represents one independent subject.

Two investigators performed the EIM recording (CHH and SBR) (Fig 2). SBR had many years of experience in performing the EIM assay. CHH had never done the assay before and he was trained on site on how to perform the assay. The training included preparation of the dog skin, placement of the EIM probe, and evaluation of the data on a display to distinguish good signals versus poor signals that were caused by technical errors. No ongoing oversight was provided outside of this basic training. A similar training approach had been used previously in the clinical setting and revealed good reproducibility [38, 47].

### Histopathology

A muscle biopsy was obtained from the central portion of the biceps femoris in a subset of normal and affected dogs after the EIM measurement (Table 1). Haematoxylin and eosin (HE) staining was used to study the general histopathology. Slides were viewed using a Nikon E800 fluorescence microscope. Photomicrographs were taken with a QImage Retiga 1300 camera. Central nucleation and the myofiber diameter were determined from ≥ 5 random microscopic fields of an HE stained muscle section. The myofiber diameter was determined by the Feret minimum diameter method using Image J (https://imagej.nih.gov/ij/docs/guide/146-30.html).

### Hydroxyproline assay

Muscle fibrosis was measured by quantifying the hydroxyproline content according to a previously published protocol [48]. Briefly, the muscle sample was hydrolyzed in 1 ml 6 N HCl for 3 h at 115°C. After neutralization with 10 N NaOH (to the final pH of 7.5), the muscle lysate was oxidized with chlormatine-T. The hydroxyproline content was quantified by measuring the color absorbance at 558 nm. The hydroxyproline concentration was determined from a standard curve calculated from a linear dilution of L-hydroxyproline (Sigma-Aldrich, Saint Louis, MO). Due to insufficient amount of muscle tissue obtained from biopsy, the hydroxyproline content in one of the study dogs (Dog ID: E09) was not measured (Table 1).

### EIM data analysis

The dog identification was coded and the data analyzed blindly. A large set of multi-frequency EIM data was collected from each subject. Data from the triplicate measurements were averaged at each frequency prior to analysis. Data from the left and right side of the same subject were similar and were averaged as a single entry in data analysis. All three electrode-array configurations yielded similar results. For the purpose of presentation, figures were drawn with the data from the long longitudinal array in this manuscript. Multi-frequency analyses were performed to show the entire spectral view of EIM values in normal and affected dogs. Selected single frequencies were further analyzed to illustrate the differences between groups (young dogs versus mature dogs and normal dogs versus affected dogs). Such frequency variation is important to assess since the myofiber size is inversely related to the peak of the reactance and phase curves. Given that dogs have not been studied with EIM to date, the frequency that is most sensitive to dystrophic alteration cannot be known a priori.

### Statistical analysis

The bar graph data were presented as mean ± standard error of mean. The multi-frequency data were presented as the population average. The myofiber diameter distribution data were presented as the percentage of the whole population. The correlation data were presented for individual subject. Statistical analysis was performed with the Matlab software (Mathworks, Natick MA). Statistical significance among multiple groups was determined by two-way ANOVA. If significance was established, post-hoc Mann-Whitney tests were performed to determine statistical significance between two groups. The relationship between the EIM data and the hydroxyproline content/myofiber diameter was established by Spearman correlation analysis using the EIM data from the subjects that had undergone biopsy. Significance was established at p<0.05.

## Results

### Overview of the study and reliability of the assay

An important goal of our study is to determine whether the EIM assay can be reliably conducted by different investigators. We compared phase data obtained by an expert evaluator and a beginner (Fig 2). The intra-class correlation coefficient (ICC) is a commonly used index for quantifying the reliability of measurements between different raters [49]. The ICC was 0.98 in our study (Fig 2A). The Bland–Altman plot illustrates dispersion of agreement by showing the magnitudes of differences in ratings in relation to the standard deviation of differences [50]. In our study, all differences were within the range of ± 2 ohms on the Bland–Altman plot irrespective of the health condition of the dogs (normal or affected) (Fig 2B). There is no indication for systematic over- or under-rating.

### Qualitative assessment of EIM multifrequency data in dystrophic and healthy dogs

**Fig 3:**
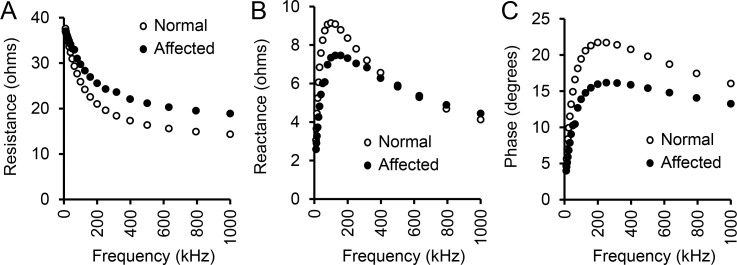
Multifrequency EIM signature in normal and affected dogs. (A) Relationship between resistance and frequency. (B) Relationship between reactance and frequency. (C) Relationship between phase and frequency. Open circle, normal dogs (n = 10); Closed circle, affected dogs (n = 14). Points represent average values across the population studied.

After establishing the robustness of the assay, we examined the overall EIM profile between normal and affected dogs cross the entire spectrum of the current frequencies from 1 kHz to 1 MHz. Similar to humans and mice, the resistance showed an exponential decline with increasing frequency in both normal and affected dogs (Fig 3A). Dystrophic and normal muscle had similar resistance at the frequency of ≤ 25 kHz. Affected dog muscle showed consistently higher resistance thereafter. With the increase in the assay frequency, the difference in resistance between dystrophic and normal muscle became more apparent and it reached a plateau of ~ 5 ohms at the frequency of ≥ 200 kHz (Fig 3A).

The overall average reactance and phase pattern showed trends similar to that observed in humans and mice (Fig 3B and 3C). The multi-frequency reactance pattern was interesting (Fig 3B). When the frequency was <500 kHz, normal muscle had a higher reactance value. The difference reached the peak at 40 to 70 kHz where the reactance of normal muscle was ~2.5 ohms higher than that of dystrophic muscle. At the frequency of 500 kHz, normal and affected dogs yielded a similar reactance of ~ 6 ohms. When the frequency was > 500 kHz, the trend appeared to have reversed. The reactance of dystrophic muscle became slightly higher than that of normal muscle (Fig 3B).

The phase value of normal muscle was higher than that of dystrophic muscle in all frequencies tested (Fig 3C). The maximal difference was seen at 100 to 200 kHz where the difference was ~ 6 degrees.

### Detailed analysis reveals statistically significant differences between normal and affected mature dogs in phase values

**Fig 4:**
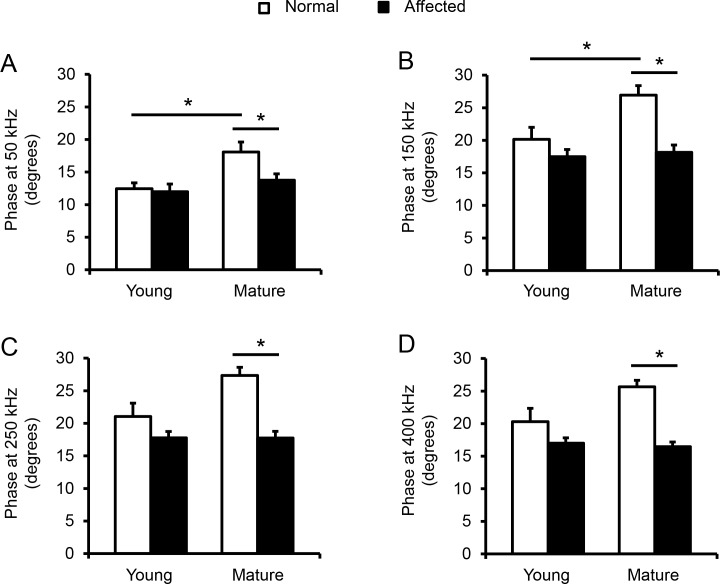
Multi-group comparison of phase. (A) Phase at 50 kHz. There was statistically significant difference between young and mature normal dogs, and between mature normal and affected dogs. (B) Phase at 150 kHz. There was a significant difference between immature young adult and mature adult normal dogs and between mature normal and affected dogs. (C) Phase at 250 kHz. There was a significant difference between mature normal and affected dogs. (D) Phase at 400 kHz. There was a significant difference between mature normal and affected dogs. Open bar, normal dogs; filled bar, affected dogs. The sample size for young normal, young affected, mature normal and mature affected dogs are 6, 8, 4 and 6, respectively. Asterisk, significantly different.

Given that most of our previous EIM studies have focused on analysis of phase values [20–28, 31–42], we specifically focused on this parameter here as well. The maximal phase was obtained at ~250 kHz for both normal and dystrophic dogs (Fig 3C), a value considerably higher than typically seen in humans. Thus, in order to identify which frequency most effectively distinguished all four groups, we compared the phase values at 250 kHz as well as two lower frequencies (50 kHz and 150 kHz) and one higher frequency (400 kHz) in all four experimental groups (Fig 4). In young dogs, the phase values of affected dogs were reduced compared to those of normal dogs at these frequencies. However, the difference did not reach statistical significance. In mature dogs, the phase values of normal dogs were significantly higher than the corresponding values of affected dogs at all four frequencies. No difference was detected between young affected dogs and mature affected dogs. At all four frequencies, the phase values of young normal dogs were lower than those of mature normal dogs. The difference was statistically significant at 50 and 150 kHz but not at 250 and 400 kHz.

### Biopsy reveals disease and age-related changes in muscle histology and fibrosis

**Fig 5:**
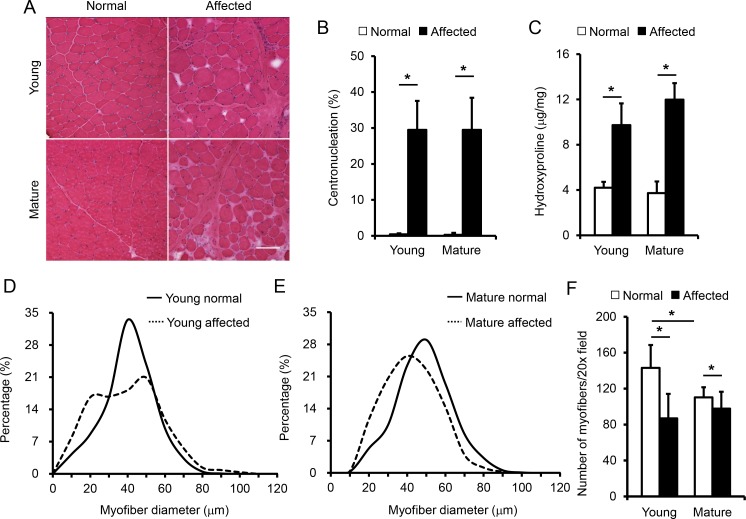
Quantitative evaluation of muscle histology and fibrosis. (A) Representative photomicrographs of the biceps femoris muscle of young normal, young affected, mature normal and mature affected dogs. Scale bar, 100 μm. Enlarged image is presented in S1 Fig. (B) Quantification of myofibers that contain centrally localized nuclei. The sample size for young normal, young affected, mature normal and mature affected groups are 4, 3, 2 and 3 dogs, respectively. There was a significant difference between normal and affected dogs in the mature age groups. (C) Quantification of muscle fibrosis by the hydroxyproline assay. The sample size for young normal, young affected, mature normal and mature affected groups are 3, 3, 2 and 3 dogs, respectively. There was a significant difference between normal and affected dogs in both age groups, but no significant difference between young and mature affected dogs. (D) Distribution of the minimum Feret diameter in young normal (n = 1,441 myofibers) and young affected (n = 1,104 myofibers) dogs. (E) Distribution of the minimum Feret diameter in mature normal (n = 1,045 myofibers) and mature affected (n = 1,225 myofibers) dogs. (F) Quantification of myofiber density per 20x filed. The numbers of 20x field counted in young normal, young affected, mature normal and mature affected dogs were 37, 30, 24 and 20, respectively. There was a significant difference between young and mature normal dogs and between normal and affected dogs. Open bar, normal dogs; filled bar, affected dogs. Asterisk, significantly different.

To correlate the EIM results with muscle disease, we biopsied the biceps femoris muscle after the EIM assay in 11 dogs including 4 young normal dogs, 3 young affected dogs, 2 mature normal dogs and 2 mature affected dogs (Table 1). On HE staining, muscle samples from normal dogs showed expected histology such as a homogenous myofiber size, peripheral localization of myonuclei, and lack of inflammatory cell infiltration and interstitial fibrosis (Fig 5A, S1 Fig). The muscle from affected dogs showed characteristic features of dystrophic pathology. Specifically, extremely large and small myofibers co-existed next to each other. In a substantial portion of myofibers, myonuclei were present at the center. There were also abundant inflammatory cells and a clear increase of interstitial tissue between muscle cells indicating muscle fibrosis (Fig 5A, S1 Fig). Nevertheless, there was no substantial difference between affected dogs at the two different ages (Fig 5A, S1 Fig).

To more accurately document muscle pathology, we quantified the percent of centrally nucleated myofibers (Fig 5B), the hydroxyproline content (Fig 5C), myofiber size distribution (Fig 5D and 5E), and muscle cell density (Fig 5F). On morphometric quantification, affected dogs showed a centronucleation of ~30% while normal dogs had < 0.5% in both age groups (Fig 5B). The hydroxyproline quantification is one of the most reliable assays to evaluate muscle fibrosis. In normal dogs, it was ~ 4 μg/mg but in affected dogs it reached ≥ 10 μg/mg (Fig 5C). Adult affected dogs had a slightly higher hydroxyproline content than young adult affected dogs but the difference was not statistically significant (Fig 5C). On fiber diameter quantification, young adult normal dogs showed a sharp bell curve indicating most of the myofibers are of a similar size (Fig 5D). Young affected dogs showed an interesting broad dual peak curve. They also had more very small and very large myofibers (Fig 5D). In adult normal dogs, the upstroke of the curve shifted toward left suggesting an absence of extremely small myofibers (Fig 5E). This correlated well with the dog growth during maturation. This was also consistent with the myofiber density quantification which showed a significant reduction of the myofiber number per 20x field in mature normal dogs compared to that of young normal dogs (Fig 5F). Compared with mature normal dogs, mature affected dogs had a broader myofiber size distribution curve and also more small myofibers (Fig 5E). It is worth pointing out that in both age groups, there were significantly more myofibers per 20x field in normal dogs compared to that of affected dogs (Fig 5F). This is consistent with our HE staining and hydroxyproline quantification suggesting there was more interstitial fibrosis in affected dogs.

### EIM changes correlates with muscle fibrosis

**Fig 6:**
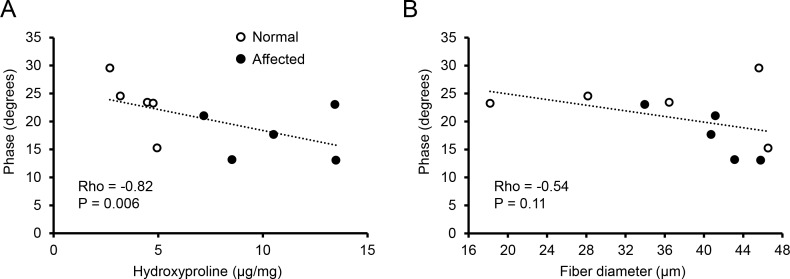
Spearman analysis of correlation between the EIM data and disease status. (A) Correlation between phase at 150 kHz and the hydroxyproline content. (B) Correlation between phase at 150 kHz and the myofiber diameter. Rho and p values are marked for each correlation analysis. Open circle, normal dogs; closed circle, affected dogs. Each circle represents one independent subject.

With quantitative data from the EIM assay and muscle pathology evaluation, we examined whether there was a correlation between these two different outcome measurements. For the purpose of analysis, we used data from phase at 150 kHz since it showed the greatest differences across groups (Fig 4). On Spearman correlation analysis, we detected a statistically significant inverse correlation between the EIM data and the hydroxyproline content (Rho = -0.82 and p = 0.006) (Fig 6A). Specifically, when the level of fibrosis was low, the phase value was high (Fig 6A). Correlation analysis between the phase and the myofiber size showed a modest relationship, which was not statistically significant (Rho = -0.54 and p = 0.11) (Fig 6B).

## Discussion

EIM has been extensively used to study neuromuscular pathologies in humans and rodents. Some studies also explored EIM in bovine muscle in vitro [51]. However, EIM has never been used in dogs. Here we present the first study applying EIM to the canine model of a neuromuscular disease. Our goals were to determine (1) whether it is easy to conduct the EIM assay in dogs and whether reproducible data can be generated, (2) whether EIM can discriminate normal and dystrophic dog muscle, (3) whether age maturation (from immature young adult to mature adult) influences the EIM results, and (4) whether the EIM data correlate with disease status determined by classical histological quantification and biochemical assays.

Consistent with our previous studies in human patients and rodent models, we found EIM measurement was easy to perform on the surface muscle of a dog limb (Fig 1). Training was straightforward. There was excellent inter-evaluator reliability between an experienced assay performer and a trainee (Fig 2) [35, 42, 52–54].

In early studies, a single 50 kHz frequency device was used to measure muscle electric impedance. Recent studies suggest that multi-frequency EIM is a better option in diagnosing normal from diseased muscle and even in distinguishing different neuromuscular diseases [26, 32, 37, 55]. By profiling three impedance parameters (resistance, reactance and phase), we obtained distinctive patterns between normal and affected dogs, suggesting EIM is a sensitive measure in the canine model of DMD (Fig 3). Comparing with our published data in amyotrophic lateral sclerosis (ALS) and inclusion-body myositis patients [19, 32], we noticed that the values of resistance were always increased but the values of phase were always reduced in diseased muscles across a spectrum of frequencies (Fig 3) [19, 32]. However, there were unique disease-specific features. For example, at low frequencies, the difference in resistance was lost between normal and affected dogs in our study but the difference persisted between normal people and patients with inclusion-body myositis throughout the entire range of frequencies (Fig 3A) [19]. Importantly, these multifrequency variations in measured phase values are mirrored in boys with DMD that have taken part in the longitudinal QED study [SBR, unpublished results] (https://clinicaltrials.gov/ct2/show/NCT01491555).

Previous studies suggested that the age influences EIM results [41, 56, 57]. Specifically, EIM parameters display an age-associated decline in phase and reactance in older individuals [56, 57] but an increase in phase and reactance in children with growth [28]. A direct comparison of EIM results of 2-m-old and 18-m-old normal mice also suggests that phase and reactance were significantly increased from 2 to 18 months [41]. Similarly, in our study, phase at 50 and 150 kHz was also significantly increased in mature adult dogs (Fig 4A and 4B). A similar trend was observed at 250 and 400 kHz though it did not reach statistical significance (Fig 4C and 4D). We suspect that the increase of the phase values in mature adult dogs of our study is likely due to muscle growth. The subjects in our young and mature normal dogs were at ~7 and ~17-m-old, respectively (Table 1). During this period, dogs are reaching their sexual maturity and still growing. In support, mature normal dogs had fewer small-size myofibers (Fig 5D and 5E) and fewer myofibers per unit area (Fig 5F), suggesting they indeed had larger myofibers.

Interestingly, we did not see much difference between young and mature affected dogs in phase (Fig 4). EIM measures muscle composition and structure. Hence, EIM results are subject to changes of many factors (such as the myofiber size, amount of fat and fibrotic tissue, and inflammation and edema). Although some differences in the pattern of myofiber size distribution were observed between young and mature affected dogs (Fig 5D and 5E), we did not see noticeable progress of muscle disease from ~7 to ~17 months by quantitative analyses of muscle pathology (centronucleation, myofiber number per unit area and hydroxyproline content). Clinically, no major differences in disease presentations were noticed between young and mature affected dogs used in this study. There was no significant difference in the body weight (Table 1). Dogs in both age groups showed similar activity. Although hypersalavation was seen in some mature affected dogs, immobilization was not observed in any affected dog in our study. Collectively, it appears that muscle disease was relatively stable from ~7 to ~17 months in affected dogs evaluated in this study. Although large-scale population studies are needed to validate this intriguing finding, our observations in affected dogs seems to mirror the so-called “honeymoon phase” in DMD patients [4, 58–61]. Hence, the lack of difference in the EIM assay agreed well with the relatively stationary disease course. To more accurately quantify the relationship between the EIM data and muscle status, we performed a Spearman correlation analysis using the reactance value at 150 kHz (Fig 6). Although there was no clear correlation between the reactance and the myofiber diameter, a statistically definitive correlation was found between the EIM data and the level of muscle fibrosis (Fig 6A). Collectively, the EIM findings appear to have reliably reflected muscle health in the context of the canine DMD model.

As a proof-of-principle study, we have demonstrated that the EIM assay is a promising technique for studying neuromuscular diseases in large animal models. However, validity studies are needed to fully establish EIM as a biomarker. Specifically, (1) we have collected a vast amount of impedance data in this study. Additional in-depth data mining is needed to identify the most sensitive parameters, including evaluation of a variety of multifrequency measures, such as multi-frequency ratios and arithmetically derived composite scores [36, 37]; (2) in this study, we only had male normal dogs. Since sex may influence EIM [57], we need to expand our study to include both male and female normal dogs; (3) for the convenience, we have only studied one dog muscle (biceps femoris) in the current study. With the further development of the technique (for example, the custom-designed EIM apparatus), we may evaluate a variety of different surface muscles to gain a more global evaluation of the disease in the dog model; (4) in this study, we have focused on correlating the pathological findings with the EIM data, there is a need to determine whether the EIM data relate well with the results of physiological assays such as muscle force measurement, gait analysis and activity monitoring in dogs [12, 17, 18]; results in mdx mice (Seward B. Rutkove, unpublished results) and ALS mice suggest a relationship between muscle force measurement and impedance values [26]. Similarly, it will be worthwhile to compare EIM with muscle ultrasound and MRI [38, 40]; (5) as a cross-sectional study, we only selected two age groups. To establish a robust natural history profile for the entire population of normal and affected dogs, we are obligated to conduct longitudinal follow-up studies on a large cohort of dogs; (6) as our ultimate goal is to develop an effective therapy for DMD, it will be necessary to implement the EIM assay in preclinical therapy studies to help quantify the efficacy of novel experimental interventions.

## Supporting information

S1 Fig This is an enlarged image of Fig 5A. (TIF)
