# Supplementary figures and images for: Non-invasive evaluation of muscle disease in the canine model of Duchenne muscular dystrophy by electrical impedance myography
Source: PLoS One. 2017 Mar 24;12(3):e0173557. doi: 10.1371/journal.pone.0173557 (PMC5365102; doi:10.1371/journal.pone.0173557)

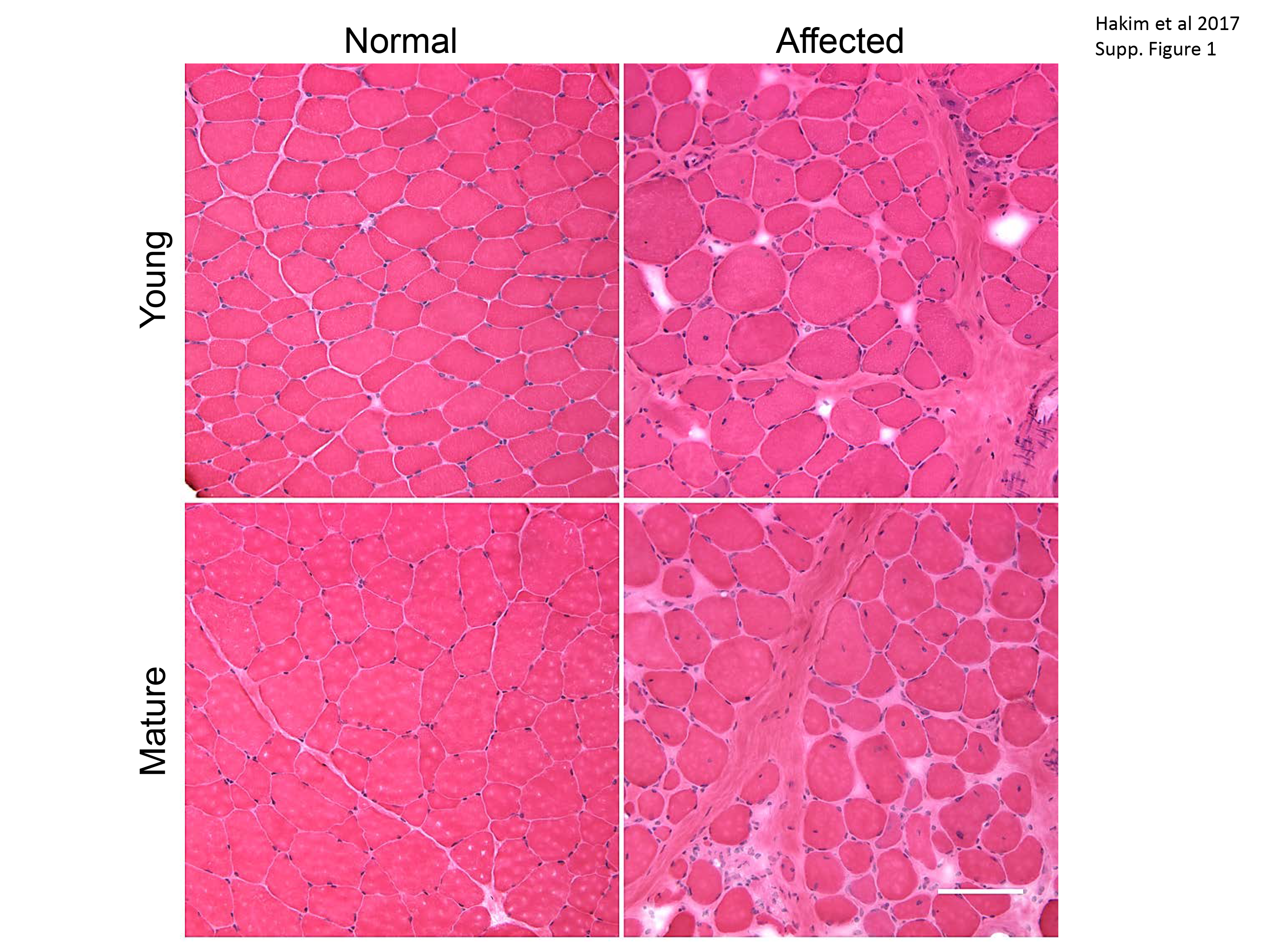

Supplement: S1 Fig — (TIF) [file pone.0173557.s001.tif]
